# Vitamin D Deficiency and Carotid Media-Intima Thickness in Childhood Cancer Survivors

**DOI:** 10.3390/nu15102333

**Published:** 2023-05-16

**Authors:** Eryk Latoch, Kacper Kozłowski, Katarzyna Konończuk, Beata Żelazowska-Rutkowska, Monika Tomczuk-Ostapczuk, Maryna Krawczuk-Rybak, Katarzyna Muszyńska-Rosłan

**Affiliations:** 1 Department of Pediatric Oncology and Hematology, Medical University of Bialystok, 15-222 Bialystok, Poland; kozlowskikacper@gmail.com (K.K.); kononczukk@gmail.com (K.K.); maryna.krawczuk-rybak@umb.edu.pl (M.K.-R.); kmroslan@post.pl (K.M.-R.); 2 Department of Pediatric Laboratory Diagnostic, Medical University of Bialystok, 15-222 Bialystok, Poland; zelazowskab@wp.pl; 3 Department of Pediatric Radiology, Medical University of Bialystok, 15-222 Bialystok, Poland; monika.tomczuk@udsk.pl

**Keywords:** atherosclerosis, cancer, CCS, children, late effects

## Abstract

Childhood cancer survivors (CCS) are predisposed to developing numerous late effects of anticancer treatment later in life. The existing literature suggests that vitamin D deficiency (VDD) may influence cardiovascular abnormalities and metabolic diseases. The objectives of this study were to investigate the prevalence of VDD among childhood cancer survivors and examine the association of vitamin D deficiency and carotid intima-media thickness (IMT). The study comprised 111 childhood cancer survivors (62 males, 49 females) with a median follow-up time of 6.14 years. Vitamin D status was determined by measuring serum 25(OH)D levels using the automatic immunoenzymatic method. Ultrasonography of the common carotid artery (CCA), the carotid bulb, and the proximal part of the internal carotid artery (ICA) was conducted. Vitamin D deficiency (<20 ng/mL) was detected in 69.4% of CCS. A higher parathormone level and increased BMI were observed among VDD survivors. No effects of type of diagnosis, radiotherapy or hematopoietic stem cell transplantation on vitamin D status were observed. Our findings reveal that survivors with VDD exhibited significantly greater thickness in the CCA and carotid bulb. In conclusion, the results of our study of childhood cancer survivors demonstrate that vitamin D deficiency is prevalent in up to 70% of individuals. We did not confirm the hypothesis that factors related to anticancer treatment used during childhood contributed to the higher prevalence of VDD. Additionally, we did not verify the contribution of vitamin D deficiency to the increase in IMT thickness.

## 1. Introduction

In recent years, significant advances in the diagnosis and treatment of childhood malignancies have led to a substantial increase in the number of survivors. Current studies indicate that this population is particularly susceptible to the earlier onset of numerous lifestyle diseases. Among the most prevalent are cardiovascular diseases, which are the leading causes of death worldwide, including heart attacks, strokes, atherosclerosis, and hypertension. Recently, it has been increasingly emphasized that in addition to the influence of genetic and environmental factors, the earlier development of many diseases as compared to the general population results from previous anticancer treatment [1,2]. An emerging population of childhood cancer survivors (CCS) is also predisposed to dyslipidemia, metabolic syndrome and subsequent cardiovascular disorders [3,4,5]. Moreover, growing evidence supports a molecular basis for premature aging in association with the use of cytostatics and radiation therapy, including processes such as pro-inflammatory cytokines production, telomere attrition, and oxidative stress, which ultimately leading to damage to various cells in the body [6,7,8].

Currently, increasing emphasis is placed on the early detection of lifestyle diseases even before the full spectrum of the clinical manifestation appears. Literature data suggest that vitamin D deficiency (VDD) may contribute to cardiovascular abnormalities and metabolic diseases [9,10,11]. Vitamin D (VD) is a steroid hormone that is converted to its active form, 25-hydroxyvitamin D (25(OH)D), which then forms a complex with the vitamin D receptor (VDR) found in numerous cells in the body [12]. Vitamin D has a pleiotropic effect on the entire body, playing a key role in bone formation, calcium and phosphorus homeostasis, immune response regulation, and cell growth, including cancer cells. Additionally, VD affects the cardiovascular system through VDR receptors, which are found in endothelial cells and cardiomyocytes, modulating their stress response.

Moreover, VDD may contribute to atherosclerosis and endothelial cell proliferation, ultimately leading to hypertension, obesity, or hyperlipidemia [13]. One of the most reliable methods for detecting and assessing the progression of atherosclerosis is the evaluation of the carotid intima-media thickness (IMT) [14]. Existing data have revealed that childhood obesity positively correlates with intima-media thickness and serves as a reliable marker of cardiovascular events [15]. Furthermore, some authors indicate that chemotherapy, radiotherapy, and male gender are the independent risk factors for greater intima-media thickness among CCS [16,17,18]. However, conflicting views have been presented on whether this treatment impacts vitamin D levels many years after the end of treatment, and if vitamin D deficiency is linked to the occurrence of late sequelae, including greater IMT [19,20,21,22]. Additionally, little is known about the association between vitamin D levels and cardiovascular abnormalities in children who have survived childhood cancer [23]. Consequently, this study aimed to investigate the prevalence of VDD among childhood cancer survivors and examine the association of vitamin D deficiency and carotid intima-media thickness.

## 2. Materials and Methods

The study included 111 childhood cancer survivors (62 males, 49 females) visiting the follow-up outpatients’ clinic at the Department of Pediatric Oncology and Hematology, Medical University of Bialystok (Poland). Patients were treated for acute lymphoblastic leukemia (54), acute myeloid leukemia (5), non-Hodgkin lymphoma (9), Hodgkin lymphoma (3), neuroblastoma (6), Wilms tumor (11), rhabdomyosarcoma (4), histiocytosis (5), and other cancers (14).

The control group consisted of 48 non-obese healthy children (27 males and 21 females), without any comorbidities such as hypertension or diabetes. This group had previously been utilized in our previous research on carotid intima-media thickness carried out in a cohort of CCS [17].

All participants were White and living at a similar latitude. None of the subjects had comorbidities that could affect vitamin D levels or calcium and phosphate metabolism. Considering the widespread non-compliance with vitamin D supplementation recommendations, only patients who were not supplemented were included in the study.

Subjects were divided based on vitamin D levels into optimal (≥30 ng/mL), suboptimal (20–29.9 ng/mL), deficient (10–19.9 ng/mL) and severely deficient (0–9.9 ng/mL) categories [24]. Body mass index was calculated as weight in kilograms divided by height in squared meters (kg/m^2^). The study group was categorized into overweight (≥85th percentile), obese (≥95th percentile), and underweight (<5th percentile) subjects, and those with normal BMI based on the OLA/OLAF growth chart adjusted for age and sex [25,26]. The waist-to-height ratio (WHtR) was calculated by dividing waist circumference by height, with 0.5 set as the norm.

Vitamin D status was determined by measuring serum 25(OH)D concentrations using the automatic immunoenzyme method with Roche’s Cobas e 411 (Hitachi High-Technologies Corporation, Tokyo, Japan). Vitamin D deficiency (VDD) was defined as serum 25(OH)D levels <20 ng/mL, according to the Institute of Medicine and updated guidelines for Central Europe [27,28].

Carotid intima-media thickness measurements were performed by two radiologists using a Voluson E8 ultrasound system (General Electric Healthcare, Milwaukee, WI, USA) equipped with a linear transducer 11 LD S/N (3–12 MHz) and a computer program for arterial analysis (Advanced 4D, INTERFACE for DICOM3). The patients assumed a supine position during the study. The transducer was positioned perpendicular to the distal surface of the arterial wall behind the sternocleidomastoid muscle. The intima-media thickness was measured as the distance between two interfaces: the hyperechogenic blood-intima line and the hypoechogenic media-adventitia line. Six segments in the right and left carotid arteries were measured: the distal part (1 cm proximal to the bulb) of the common carotid artery (CCA), the carotid bulb, and the proximal part of the internal carotid artery (ICA). At each site, three measurements were taken, and the median value was used as the score. In most patients, the intima-media layer exhibited linear continuity. When discontinuity occurred, the intima-media thickness was measured on the linear part of the artery. No atherosclerosis, microcalcifications, fatty streaks, or granulations were observed on the arteries.

Statistical analysis was performed using STATA version 13.3. Distribution was assessed with the Shapiro–Wilk test. Data were expressed as median (M) and quartiles (Q) or mean and standard deviation (SD) when appropriate. The Student t-test or Mann–Whitney U test was used to compare the continuous variables. Univariate analysis of variance was performed using ANOVA, and post hoc analysis was conducted with Tukey’s test. The χ^2^ test was used to compare numerical variables. Correlations between parameters were evaluated using Spearman’s rank correlation coefficient. Multivariate regression models were employed to examine the association between 25(OH)D and the independent variables that might potentially affect their level. A *p*-value of <0.05 was considered statistically significant.

## 3. Results

The characteristics of childhood cancer survivors are shown in Table 1. The median age at diagnosis was 4.31 years (range: 1 month–17.5 years), and the median time elapsed since the end of treatment was 6.14 years (range: 1.55–13.47). The study group comprised 111 CCS compared to 48 age- (13.45 vs. 15.0, *p* = 0.195) and sex-matched (M/F: 62/49 vs. 27/21, *p* = 0.965) controls. No statistically significant differences were observed between the study and control groups in body weight (50.70 kg (range 38.00–66.00) vs. 54.50 kg (range 45.00–63.50), *p* = 0.704), height (1.62 m (range 1.44–1.69) vs. 1.60 m (range 1.55–1.70), *p* = 0.085), or BMI (20.20 kg/m^2^ (range 17.73–24.05) vs. 20.70 kg/m^2^ (range 19.12–22.91), *p* = 0.858).

The median 25(OH)D serum concentration in the entire study group was 16 ng/mL (range 10.49–23.00) compared to 18.65 ng/mL (range 13.00–25.10) in the control group (*p* = 0.132). No differences were found in 25(OH)D serum concentration according to sex in the study group (female: 14 ng/mL (range 10.95–20) vs. male: 17.00 ng/mL (range 10.49–24.00) *p* = 0.426) or in the control group (female: 18.30 ng/mL (range 11.50–21.00); male: 19.00 ng/mL (15.13–26.00), *p* = 0.164). Additionally, no significant gender differences were observed compared to the control group (*p* > 0.05).

No correlations between type of diagnosis and vitamin D concentrations were found. The mean serum 25(OH)D levels in leukemia, lymphoma, and solid tumor groups were 14.00 ng/mL (range 10.00–23.22), 19.30 ng/mL (range 9.02–22.50), and 16.00 ng/mL (range 11.78–24.00), respectively (*p* = 0.79). Neither past radiotherapy use nor the HSCT procedure influenced vitamin D levels (*p* < 0.05).

The study participants were divided into four subsets according to their 25(OH)D level ranges (Table 2). Vitamin D deficiency (VDD) was found in 77 children (69.4%). Table 3 displays the characteristics of CCS based on serum 25(OH)D levels (below and above 20 ng/mL). Compared to patients with 25(OH)D levels >20 ng/mL, survivors with vitamin D deficiency presented significantly higher body weight (53.70 kg (range 44.30–71.00) vs. 40.85 kg (range 34.35–55.60), *p* = 0.014), BMI (20.57 kg/m^2^ (range 18.52–25.46) vs. 18.23 kg/m^2^ (range 16.93–22.15), *p* = 0.038), and parathormone level (35.40 pg/mL (11.50; 101) vs. 26.45 pg/mL (12.10; 55.80), *p* = 0.011). Furthermore, the IMT analysis revealed a statistically significantly greater thickness of the right CCA, and the CCA and bulb on the left side, among individuals with 25(OH)D levels above 20 ng/mL.

Our further analysis investigated the relationship between 25(OH)D serum concentrations and carotid intima-media thickness (Table 4). There was a strong positive correlation between vitamin D and CCA on the right (*r* = 0.61, *p* < 0.001) and a moderate correlation with that on the left (*r* = 0.55, *p* < 0.001) side. Additionally, a moderate positive correlation was observed between 25(OH)D serum concentrations and the bulb region on both the right (*r* = 0.44, *p* = 0.006) and left (*r* = 0.47, *p* = 0.003) sides. Likewise, ICA IMT positively correlated with vitamin D levels on the right (*r* = 0.43, *p* = 0.007) and left (*r* = 0.42, *p* = 0.009) sides.

In the entire study group, 42 normal weight, 7 overweight, and 15 obese survivors were identified. The mean vitamin D levels were 16 ng/mL (range 11.00–25.00) for normal weight, 17.00 ng/mL (range 9.84–20.00) for overweight, and 11.00 ng/mL (range 9.00–23.22) for obese participants, respectively (*p* = 0.707).

A multiple regression model was developed to examine each variable that might affect the serum level of 25(OH)D. The analysis included potential confounding anthropometric and biochemical variables, listed in Table 3. The created model revealed that, out of all factors introduced in the model, only BMI and parathormone levels significantly affected 25(OH)D concentrations (coeff. −0.56 and −0.15, respectively; *p* < 0.05).

## 4. Discussion

At present, in addition to achieving the highest possible cure rate, one of the major challenges in pediatric oncology is the prevention and early detection of the late sequelae resulting from childhood cancer treatment. While much is already known about the impact of risk factors directly related to treatment, it remains unclear what other factors contribute to the higher incidence of lifestyle diseases occurring earlier in life among CCSs. Numerous markers for the development of these diseases remain undiscovered. One postulated trigger is the role of vitamin D deficiency in the emergence of long-term sequelae many years after the cessation of treatment.

Numerous large epidemiological studies have demonstrated that vitamin D deficiency in children is highly prevalent worldwide [28]. This observation is supported by the current study, which shows a significant deficiency in patients after childhood cancer treatment. The percentage of VDD among the subjects was 69.4% and did not differ from that in the control group. Existing data in this field have indicated that the prevalence of VDD among CCSs ranges from 25 to 90%, as confirmed by our analysis [19,29,30,31]. Various potential factors affecting the incidence of VDD in published studies were analyzed. For instance, Bhandari et al. found VDD in 25% of participants, whereas multivariable analysis showed that higher BMI among CCSs was associated with higher odds of VDD (overweight: OR 1.78; obese: OR 2.40) [19]. This may be a result of the fact that high body mass contributes to the incidence of vitamin D deficiency, most likely through the enhanced sequestration of vitamin D in adipose tissue. This observation, however, should be confirmed by more accurate anthropometric measurements, such as the amount of body fat mass or water, which was not the focus of this study and requires further analysis. In addition, it should be noted that in the present study, most patients were of normal weight (80%). Although we have shown a positive association between BMI and VDD, we should be cautious in drawing firm conclusions about weight reductions if they are within the normal range for age and gender. Choudhary et al., in a study conducted on 484 CCSs, showed that 29% of them presented VDD. In turn, univariate analysis revealed that race and age at cancer diagnosis were associated with 25(OH)D insufficiency [29]. However, we did not confirm these links in our report. In a retrospective study by Rosen et al., 53.7% of cancer survivors had vitamin D deficiency, and this decreased over time. Patients with solid tumors were the most affected, despite their lack of routine exposure to glucocorticoids [31]. Notably, these subgroups were very small and there was no comparison with healthy controls. We did not confirm the link between the type of diagnosis and VDD in our analysis, which is in line with the mentioned studies. Nor did we find a detrimental effect of radiotherapy or HSCT procedure on the incidence of VDD. Another retrospective cross-sectional study showed 25(OH)D levels <30 ng/mL in 89.7% of CCSs [30]. The authors emphasized that pediatric cancer patients require frequent monitoring of 25(OH)D levels, and better results were obtained with supplementation with higher doses of vitamin D for longer periods of time. Most papers, but not all, indicate the high prevalence of VVD among CCS. However, in a U.S. study by Esbenshade et al., a high prevalence of VDD was found in only 15.8% and vitamin D insufficiency (VDI) in 34.5% of cancer survivors, while VDD/VDI combined was associated with higher BMI (OR = 5.4), older age (OR = 2.2), non-Caucasian or Hispanic race (OR = 4.5) and summer versus winter season (OR = 0.12) [32]. These results confirm the association between obesity and low vitamin D levels also noted in the general population.

Numerous studies have reported on the relationship between vitamin D status and carotid intima-media thickness; however, the available studies among children, including CCS, are severely limited [23,33,34]. Most, but not all, reports to date indicate an increased intima-media complex in patients with VDD. Two meta-analyses have shown an inverse relationship between vitamin D status and carotid intima-media thickness [35,36]. Nevertheless, the cause-and-effect relationship is still not fully understood. Contrary to our expectations, the main finding of our study was the positive correlation between serum 25(OH)D levels and carotid intima-media thickness—higher vitamin D levels were observed in patients with greater intima-media complex thickness. The parameters significantly associated with vitamin D status were the thickness of the common carotid arteries and the carotid bulbs. Among the few studies in children addressing this issue, we only identified papers conducted among infants and obese adolescents [37,38,39]. No significant correlation was found between vitamin D and aortic or carotid intima-media thickness in term infants [39]. In turn, papers focusing on obese adolescents provide conflicting results. In a cross-sectional study by Murini et al. on obese children aged 15 to 17 years, no association between VDD and IMT was noticed [38]. In contrast, a study by Ashgari et al. in girls and overweight individuals found no significant association of 25(OH)D and iPTH/25(OH)D ratio with IMT; however, high iPTH levels and iPTH/25(OH)D ratio were associated with increased IMT in boys and obese subjects [37]. In our study, higher PTH levels were observed in VDD patients, but no linkage to IMT was established.

A review of the literature revealed limited data on the vitamin D status and IMT relationship in CCS, with only one paper addressing this issue. A study conducted by Muggeo et al. on a group of 52 ALL survivors showed a higher prevalence of 25(OH)D deficiency than in controls. In a univariate analysis, VDD patients demonstrated higher IMT values compared to those with a normal range of vitamin D, which is in contrast to our observations [23]. These discrepancies may have arisen since our study enrolled individuals with various types of cancers, not just with ALL. Treatment of acute lymphoblastic leukemia involves the use of high doses of systemic steroids, which can lead to impaired adipose tissue development and thus vitamin D deficits later in life. Another point that should be addressed is the lack of reliable studies on IMT references in pediatrics, making it difficult to diagnose early vascular disease during childhood based solely on IMT measurement.

There are several limitations of this study. The cross-sectional design cannot clearly explain causal pathways, and the single-center analysis should be interpreted within the context of possible biases that also may occur. The heterogeneity of the study group with respect to different cancer types made it unfeasible to analyze the impacts of some specific therapeutic protocols. Due to significant technical issues, it was not possible to assess the skeletal status by measuring bone mineral density. It is also worth highlighting that pubertal period might also influence both IMT and VD, which was not assessed in this study. The added value of the study could be represented by a comparison of the results with a reference group of obese children, which was not investigated in this analysis. Due to significant limitations, the effect of sunlight exposure could not be studied.

The strengths of our research include a relatively long follow-up time and no ethnic diversity. To the best of our knowledge, apart from one published study in patients with ALL, this is the first study to evaluate the association of vitamin D status and intima-media thickness in childhood cancer survivors with different types of cancer.

In conclusion, the results of our cross-sectional study of childhood cancer survivors demonstrate that vitamin D deficiency is widespread, affecting approximately 70% of individuals. Among the many factors studied, higher parathormone levels, high body weight and increased BMI were observed among CCSs with VDD, which supports the results of other studies conducted to date. Yet these values were within the normal range for most CCSs, so it would be relevant to conduct a longitudinal observational study on large cohorts of CCSs with abnormal body weights to confirm whether overweight and obese CCSs are at risk for low vitamin D levels. No link was observed between type of diagnosis, radiotherapy or hematopoietic stem cell transplantation and vitamin D status. Consequently, we could not confirm the hypothesis that factors related to anticancer treatment used in childhood affect the higher prevalence of VDD. Another key finding was higher serum 25(OH)D levels in patients with greater intima-media complex thickness, which did not confirm the contribution of vitamin D deficiency to increased IMT thickness. Finally, we would like to highlight two essential issues. The first relates to adherence to vitamin D supplementation, which seems crucial for maintaining normal vitamin D levels and the functioning of many systems and organs in CCS [40]. Secondly, considering the studies conducted in adult patients and the proven higher risk of cardiovascular disease among childhood cancer survivors, further prospective longitudinal studies evaluating carotid intima-media thickness and vitamin D status should be conducted on a large CCSs cohort.

## Figures and Tables

**Table 1:** Clinical characteristics of the childhood cancer survivors (CSSs).

	Total	Male	Female
Patients (*n*, %)	111 (100%)	62 (55.9%)	49 (44.1%)
Age at diagnosis (years)	4.31 (2.93; 6.97)	4.58 (3.17; 7.53)	4.23 (2.88; 6.28)
Age at study (years)	13.45 (11.08; 16.8)	14.03 (11.20; 17.64)	13.13 (11.04; 14.53)
Follow-up time (years)	6.14 (4.5; 9.1)	6.78 (5.06; 9.79)	5.76 (4.12; 8.18)
Weight (kg)	52.98 (17.5; 98)	54.56 (17.50; 98.00)	51.35 (23.50; 81.00)
Height (m)	1.56 (1.12; 1.86)	1.59 (1.12; 1.86)	1.53 (1.23; 1.75)
BMI (kg/m^2^)	21.01 (12.62; 31.63)	21.06 (12.62; 31.64)	21.15 (14.57; 30.49)
Overweight (*n*)	7 (6.3%)	4 (3.6%)	3 (2.7%)
Obese (*n*)	15 (13.5%)	7 (6.3%)	8 (7.2%)
Underweight (*n*)	2 (1.8%)	2 (1.8%)	0
WHtR > 0.5 (*n*)	17 (15.3%)	10 (9%)	7 (6.3%)
Diagnosis (*n*, %)	111 (100%)	62 (55.9%)	49 (44.1%)
Acute leukemia	59 (53.1%)	32 (28.8%)	27 (24.3%)
Lymphoma	12 (10.8%)	9 (8.1%)	3 (2.7%)
Solid tumors	40 (36.1%)	21 (19%)	19 (17.1%)
Radiotherapy	17 (15.3%)	10 (9%)	7 (6.3%)
HSCT	9 (8.1%)	6 (5.4%)	3 (2.7%)

Data are given as number (*n*), percentage, and median and interquartile range (Q1; Q3). WHtR: waist-to-height ratio; HSCT: hematopoietic stem cell transplantation; BMI: Body Mass Index.

**Table 2:** Characteristics of patients in the study group according to 25(OH)D levels.

	Childhood Cancer Survivors (*n* = 111)	Control Group (*n* = 48)	*p* Value
Severe Deficiency (0–9.9 ng/mL)	22 (19.8%)	7 (15%)	0.432
Deficiency (10–19.9 ng/mL)	55 (49.5%)	22 (46%)	0.666
Suboptimal Concentration (20–29.9 ng/mL)	23 (20.7%)	15 (31%)	0.152
Optimal Concentration (≥30 ng/mL)	11 (10%)	4 (8%)	0.755

Data are given as number (*n*) and percentage.

**Table 3:** Clinical and biochemical characteristics of childhood cancer survivors (CCS) according to 25(OH)D level.

Variables	25(OH)D		*p* Value
	<20 ng/mL	≥20 ng/mL	
Number of patients (*n*, %)	77 (69.4%)	34 (30.6%)	-
Male/Female (*n*/*n*)	40/37	22/12	0.422
Age at diagnosis (years)	4.4 (2.8; 6.9)	4.3 (3.11; 6.9)	0.972
Age at study (years)	13.65 (11.4; 16.8)	13.2 (9.75; 16.4)	0.573
Follow-up time (years)	6.04 (3.9; 9.3)	6.45 (4.78; 8.59)	0.905
Height (m)	1.63 (1.46; 1.7)	1.51 (1.42; 1.62)	0.052
Weight (kg)	53.7 (44.3; 71)	40.85 (34.35; 55.6)	0.014
BMI (kg/m^2^)	20.6 (18.52; 25.46)	18.24 (16.94; 22.15)	0.038
WHtR	0.45 (0.42; 0.51)	0.43 (0.41; 0.46)	0.261
**Biochemical parameteres**			
25(OH)D (ng/mL)	12.00 (2.00; 20.00)	27.38 (21.00; 47.00)	0.001
Total cholesterol (mg/dL)	164.5 (145.5; 174.5)	157 (139; 181)	0.728
HDL cholesterol (mg/dL)	52 (46; 63)	54 (49; 65)	0.300
LDL cholesterol (mg/dL)	91 (72; 101)	80 (69; 92)	0.207
Triglycerides (mg/dL)	81 (58; 111)	83 (56; 105)	0.868
TSH (mU/L)	2.31 (1.76; 3.24)	2.1 (1.54; 3.27)	0.681
fT3 (ng/L)	3.99 (2.24; 5.17)	3.77 (3.20; 5.38)	0.556
fT4 (ng/L)	1.22 (0.87; 1.99)	1.26 (0.96; 2.09)	1.000
PTH (pg/mL)	35.40 (11.50; 101)	26.45 (12.10; 55.80)	0.011
Ca (mmol/L)	2.47 (2.26; 2.71)	2.48 (2.26; 2.66)	0.833
P (mg/dL)	3.93 (3.57; 4.95)	4.23 (3.43; 4.77)	0.546
ALP (U/L)	224.00 (3.18; 552.00)	292.50 (73.00; 467.00)	0.091
**Intima-media thickness**			
CCA right (mm)	0.40 (0.34; 0.44)	0.46 (0.43; 0.47)	0.001
Bulb right (mm)	0.39 (0.33, 0.44)	0.44 (0.39; 0.47)	0.063
ICA right (mm)	0.38 (0.34; 0.42)	0.42 (0.37; 0.49)	0.062
CCA left (mm)	0.38 (0.34; 0.44)	0.44 (0.40; 0.49)	0.028
Bulb left (mm)	0.36 (0.34; 0.41)	0.43 (0.38; 0.51)	0.010
ICA left (mm)	0.38 (0.35; 0.43)	0.40 (0.38; 0.46)	0.174

Data are presented as the number (*n*), percentage, and median and interquartile range (Q1; Q3). BMI: Body Mass Index; WHtR: waist-to-height ratio; TSH: thyrotropin; fT3: triiodothyronine; fT4: thyroxine; PTH: parathormone; ALP: alkaline phosphatase; CCA: common carotid artery; ICA: internal carotid artery.

**Table 4:** Univariate correlations between 25(OH)D serum level and carotid intima-media thickness.

	*r*	*p*
CCA right (mm)	0.61	<0.001
Bulb right (mm)	0.44	0.006
ICA right (mm)	0.43	0.007
CCA left (mm)	0.55	<0.001
Bulb left (mm)	0.47	0.003
ICA left (mm)	0.42	0.009

CCA: common carotid artery; ICA: internal carotid artery.

## Data Availability

Not applicable.
